# Incidental syringomatous proliferation: A benign, subclinical finding during Mohs micrographic surgery

**DOI:** 10.1016/j.jdcr.2021.06.019

**Published:** 2021-07-02

**Authors:** Maria Sarah Bovenberg, Maxwell A. Fung, Maija Kiuru, Jayne S. Joo

**Affiliations:** a Department of Dermatology, University of California Davis School of Medicine, Sacramento, California; b Department of Pathology and Laboratory Medicine, University of California Davis School of Medicine, Sacramento, California

**Keywords:** basal cell carcinoma, benign syringomatous proliferations, dermatopathology, Mohs micrographic surgery, syringoma, BCC, basal cell carcinoma, MMS, Mohs micrographic surgery

## Introduction

Mohs micrographic surgery (MMS) is the treatment of choice for basal cell carcinoma (BCC) involving the facial, acral, and genital areas due to high cure rates and tissue sparing qualities. When interpreting frozen sections of the excised tissue, incidental benign neoplasms can be encountered and sometimes complicates margin evaluation and management of the primary tumor. Here, we report a case of an incidental benign syringomatous proliferation encountered during MMS for a nodular and infiltrative BCC on the cheek. The possible occurrence of an incidental subclinical syringomatous proliferation is an important observation for Mohs surgeons to avoid excessive surgery beyond adequate resection of the primary tumor.

## Case report

**Fig 1:**
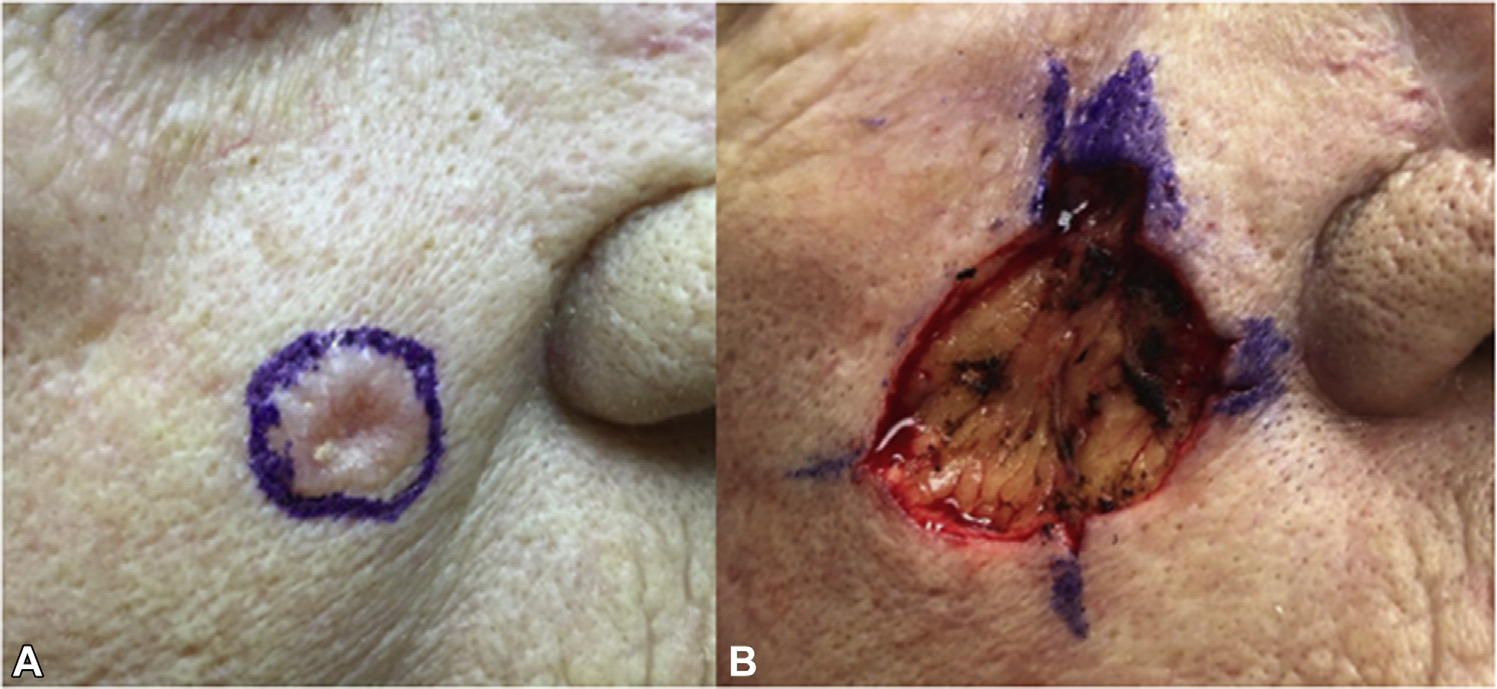
**A**, Basal cell carcinoma on the right cheek prior to biopsy and (**B**) defect after Mohs micrographic surgery. No other clinical lesions were visible on the surrounding skin.

**Fig 2:**
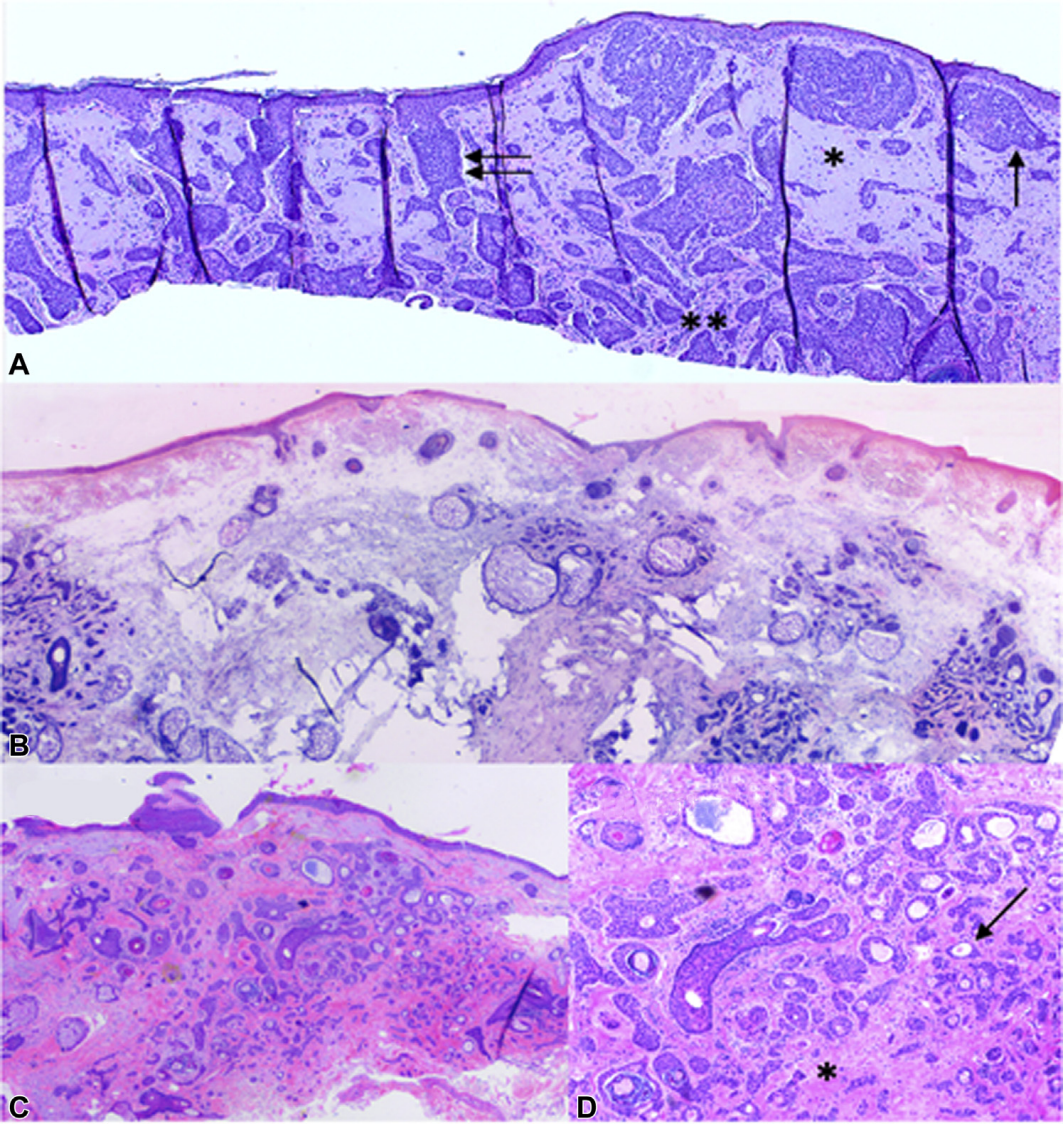
**A**, Histopathologic examination of the biopsied lesion was consistent with basal cell carcinoma with both nodular and infiltrative growth patterns. Note the characteristic features of basal cell carcinoma, including extensive background solar elastosis (*asterisk*), fibromyxoid stroma (*double asterisks*), peripheral palisading (*arrow*), and stromal retraction (*double arrows*) around the tumor islands. **B**, Mohs surgery frozen section: small islands of basaloid cells, some of which form duct-like structures, involving the papillary and mid dermis. **C**, Permanent sections: nests of basaloid cells with central ductal differentiation in “paisley tie” pattern. **D**, Higher magnification demonstrated ducts with tadpole-like structures (*arrow*) and sclerotic stroma (*asterisk*). Compared with Figure 2, *A*, there is a notable absence of cellular atypia, peripheral palisading, and stromal retraction. (**A** and **D**, Hematoxylin-eosin stain; original magnifications: **A**, ×4; **B**, ×4; **D**, ×10.)

A 79-year-old woman was referred for MMS of a biopsy-proven BCC on the right cheek. A careful physical examination at the time of her biopsy revealed a 1-cm pearly pink papule with rolled borders and central atrophy on the right cheek. No other growths were noted in the immediate vicinity of the site (Fig 1, *A*). Histopathologic examination of the biopsied lesion was consistent with BCC with both nodular and infiltrative growth patterns (Fig 2, *A*). The patient was referred for MMS given the tumor size, location, and aggressive tumor histology. The primary Mohs layer revealed 2 focal areas with small irregular folliculocentric islands of basaloid cells initially thought to be infiltrative BCC. The Mohs map was marked accordingly, and the second layer was taken from the areas of concern. The above process was repeated 2 more times. On the third layer, more foci of basaloid cells forming duct-like structures involving the papillary and mid dermis were encountered. At this stage, it became clear that these proliferative foci had a more definite syringomatous appearance and were much more widespread than in the previous layers (Fig 2, *B*). They did not extend beyond the dermal plane or exhibit perineural invasion. No cellular atypia was noted. As such, microcystic adnexal carcinoma or remaining infiltrative BCC was not favored. Upon careful intraoperative examination of the patient, no clinical lesion was visible that would correlate with the findings seen microscopically. As this proliferation was subclinical and displayed benign histopathologic features suggestive of a folliculocentric syringoma-like hamartoma, which was confirmed by an intraoperative consultation with our dermatopathologist, further resection was not performed (Fig 1, *B*). The defect was repaired primarily. The last Mohs layer was thawed and sent to the dermatopathology department for permanent histologic sections for further analysis. Evaluation of hematoxylin-eosin–stained tissue revealed numerous small ducts and epithelial nests and strands surrounded by sclerotic stroma involving the papillary and mid dermis, confirming the diagnosis of a syringomatous proliferation (Fig 2, *C* and *D*).

## Discussion

Syringomas or syringomatous proliferations are benign adnexal neoplasms of likely eccrine origin that present clinically as multiple skin-colored papules on the periorbital skin or upper trunk. They are most commonly encountered in middle-aged women.^1^^,^^2^ Histologically, they are characterized by well-circumscribed strands of basaloid cells, some of which display ductal differentiation. When sectioned at an angle, the ducts can resemble tadpoles. The proliferation is typically limited to the papillary to mid dermis, and the associated stroma is often fibrotic or sclerotic. The histopathologic differential diagnosis includes other tumors in the “paisley pattern” category, such as desmoplastic trichoepithelioma, infiltrative and morpheaform BCC, microcytic adnexal carcinoma, and sclerosing sweat duct carcinoma.^3^^,^^4^ Careful differentiation between these entities is required to avoid misdiagnosis resulting in either over- or under-treatment, especially when considering that these lesions often occur in cosmetically sensitive areas. In our patient, what made the case challenging was the subclinical presentation, and a thorough evaluation of the frozen sections was required to determine appropriate management during the patient's Mohs procedure.

In contrast to morpheaform or infiltrative BCCs, syringomas lack stromal retraction or cellular atypia. The surrounding stroma is often densely red and sclerotic rather than fibromyxoid, as seen in BCC, although sclerosis can be seen in morpheaform (sclerosing) BCCs. Deep involvement of the reticular dermis or subcutis as well as perineural invasion is incompatible with a diagnosis of syringoma and should direct the surgeon toward one of the other entities mentioned above. When doubt exists about the origin of the lesion, permanent sections should be obtained. Helpful stains to differentiate between BCC and syringoma include epithelial membrane antigen and carcinoembryonic antigen (both positive in syringoma and negative in BCC) and Ber-EP4 (positive in BCC and negative in syringoma).^1^ In our case, the histopathologic pattern of the lesion was recognizable as a benign syringomatous proliferation on hematoxylin-eosin staining, thus no additional staining was performed.

In conclusion, we report a case of a subclinical syringomatous proliferation associated with BCC discovered incidentally during Mohs surgery. Although only a few cases of single^1^, ^2^, ^3^ and multifocal^4^ benign subclinical syringomatous proliferations associated with BCC, as well as microcystic adnexal carcinoma,^5^^,^^6^ have been published, we believe that this is likely an underreported finding, as both syringomas and BCCs are relatively common neoplasms and occur on overlapping locations on the face. Awareness of these incidental benign growths and how to properly differentiate these from BCC is paramount for Mohs surgeons to avoid excessive surgery beyond adequate resection of the primary tumor.

## Conflicts of interest

None disclosed.
